# Supplementation with complex milk lipids during brain development promotes neuroplasticity without altering myelination or vascular density

**DOI:** 10.3402/fnr.v59.25765

**Published:** 2015-03-27

**Authors:** Rosamond B. Guillermo, Panzao Yang, Mark H. Vickers, Paul McJarrow, Jian Guan

**Affiliations:** 1 Liggins Institute, The University of Auckland, Auckland, New Zealand; 2 Centre for Brain Research, Faculty of Medical and Health Sciences, The University of Auckland, Auckland, New Zealand; 3 Fonterra Research and Development Centre, Palmerston North, New Zealand

**Keywords:** complex milk lipids, brain development, neuroplasticity, dopamine, myelination, vascular density

## Abstract

**Background:**

Supplementation with complex milk lipids (CML) during postnatal brain development has been shown to improve spatial reference learning in rats.

**Objective:**

The current study examined histo-biological changes in the brain following CML supplementation and their relationship to the observed improvements in memory.

**Design:**

The study used the brain tissues from the rats (male Wistar, 80 days of age) after supplementing with either CML or vehicle during postnatal day 10–80. Immunohistochemical staining of synaptophysin, glutamate receptor-1, myelin basic protein, isolectin B-4, and glial fibrillary acidic protein was performed. The average area and the density of the staining and the numbers of astrocytes and capillaries were assessed and analysed.

**Results:**

Compared with control rats, CML supplementation increased the average area of synaptophysin staining and the number of GFAP astrocytes in the CA3 sub-region of the hippocampus (*p*<0.01), but not in the CA4 sub-region. The supplementation also led to an increase in dopamine output in the striatum that was related to nigral dopamine expression (*p*<0.05), but did not alter glutamate receptors, myelination or vascular density.

**Conclusion:**

CML supplementation may enhance neuroplasticity in the CA3 sub-regions of the hippocampus. The brain regions-specific increase of astrocyte may indicate a supporting role for GFAP in synaptic plasticity. CML supplementation did not associate with postnatal white matter development or vascular remodelling.

Postnatal brain development involves many processes that are collectively important for brain function in adult life (1). While neurogenesis and migration are primary drivers of brain development before birth, synaptogenesis, myelination, and vascular remodelling are the dominant processes underpinning brain remodelling after birth (1–3). A number of novel components that may provide biological benefits beyond basic nutrition, for example, milk-derived complex lipids (including gangliosides and phospholipids), have been suggested to benefit brain development during early life and cognitive function in adulthood (4, 5). Using the Morris Water Maze (MWM) tests, we have previously shown that long-term supplementation with complex milk lipids (CML) from neonatal day 10 until postnatal day 80 (5), but not maternal supplementation during pregnancy and lactation (6), moderately improves the ability of spatial reference learning in adult rats. The differential effects on cognitive function could be due to the window of opportunity of supplementation and may suggest that the beneficial effect of dairy lipids is specific to postnatal brain development. It is therefore important to understand the biological changes associated with the improved brain function after CML supplementation.

The hippocampus and the striatum are the brain regions that process spatial reference learning and memory (7–10). The changes in synaptic plasticity in these brain regions, for example, dopamine outputs and glutamate receptors, may be the biological modifications underlying the observed improvements in learning and memory (8, 9, 11). Synaptophysin is a marker for synaptic vesicles and has been commonly used as a marker for synaptic plasticity at pre-synaptic location (12, 13). Improved dopamine neurotransmission has been reported to be associated with improved memory in aged rats after long-term supplementation with a concentrate of dairy lipids (12). In addition to a supporting role for astrocytes in neuronal and synaptic plasticity (14), neuronal–glial–vascular networking plays an essential role in neuronal function (15) and myelination (16). Phospholipids are also important components of white matter development (17) and supplementation with lipids in early life may promote myelination (18).

The current study evaluated the effect of postnatal supplementation with CML on synaptic plasticity, myelination, astrocytes, and vascular remodelling using the brain tissue collected from a previous study that demonstrated the efficacy of CML in improved memory.

## Methods

### Animal model

The brain tissues that are utilised for the current study were banked from a previous experimental study undertaken in the rat, which has been described elsewhere (5).

In brief, male Wistar rats (*n*=16 per group) were supplemented from postnatal day 10 until day 80 with either CML (a complex lipid ingredient enriched in gangliosides and phospholipids at 1% w/w of food intake) or vehicle by oral gavage before weaning (day 21) or gelatine formulated CML supplementation to a standard chow diet (Diet 2018, Harlan, Oxon) after weaning. The animals then underwent MWM tasks at the end of study. Supplementation with CML resulted in significant improvements in spatial reference memory of adult rats (5). The rats were killed at day 80 for tissue collections. Animal experiments were approved by the Animal Ethics Committee, the University of Auckland.

### Brain tissue preparations

Methods for brain tissue collection and preparation have been described previously (8). Briefly, rats were deeply anaesthetised using pentobarbital (125 mg/kg, i.p.). The brains were transcardially perfused with saline and then with 4% paraformaldehyde. The brains were removed and further fixed in the same fixatives for 48 h before being processed and embedded in paraffin. The brain sections that contained the striatum and the hippocampus (Anterior–Posterior 4.2–4.5 mm) were collected from either the CML-supplemented group (CML, *n*=16) or the vehicle-supplemented group (control, *n*=16). At least three coronal sections (8.0 µm thickness) from each brain region were used for immunohistochemical staining.

### Immunohistochemistry

The protocol for immunostaining has been described previously (9). Briefly, sections were deparaffinised in xylene, rehydrated in serial concentrations of ethanol, and washed in phosphate-buffered saline (PBS). The sections were pre-treated with 1% hydrogen peroxide in 50% methanol and then blocked with 5% normal horse serum for 1 h at room temperature. Heat-mediated antigen retrieval in citrate buffer (pH 6.0) was conducted for synaptophysin and glutamate receptor-1 (GluR-1). The following primary antibodies were used: monoclonal mouse anti-synaptophysin for synaptic vesicles (Sigma *1*:1000), rabbit anti-tyrosine hydroxylase for dopamine (TH, Protos Biotech Corporation 1:250), rabbit anti-GluR-1 for glutamate receptors (Chemicon 1:100), mouse anti-myelin basic protein for myelin density (MBP, Jomar Millipore 1:1000), and mouse anti-glial fibrillary acidic protein for astrocyte (GFAP, Sigma, 1:2000). The sections were incubated with the primary antibodies for 48 h at 4°C, and then incubated with goat anti-rabbit or goat anti-mouse secondary antibody (Sigma 1:200) accordingly overnight at 4°C before being incubated with tertiary antibody (ExtrAvidin Peroxidase 1:200) for 3 h at room temperature. The staining was visualised using 3, 3, diaminobenzidine tetrahydrochoride (DAB). For visualising blood vessels, biotin-conjugated isolectin B-4 (IB4) (Sigma 1:4000) was used (Sigma, St. Louis, MO, USA). The sections were pre-treated with 1% hydrogen peroxide in 50% methanol after being deparaffinised, and were then incubated overnight at 4°C with IB4 (1:4) in Tris-buffered saline before being developed in DAB. The sections were washed three times with PBS for 15 min between each step. Finally, the sections were washed in PBS and dehydrated in serial concentrations of ethanol and xylene before being cover-slipped with DPX.

### Image acquisition and analysis

The methods of image acquisition and analysis have been reported previously (8, 19). Briefly, light microscopy (Nikon 800, Tokyo, Japan) and photographic software (Nikon Digital Sight) were used to acquire images. At least three images from each brain region and each of three sections were analysed using image analysis (Image J or SigmaScan Pro 5.5, SPSS) (8). The average areas and densities of synaptophysin, TH, GluR-1, and MBP staining in the hippocampus, the striatum, and the substantia nigra pars compacta (SNc) were analysed. The average number and the total length of blood vessels in the hippocampus and the striatum were also analysed (19). The numbers of GFAP-positive astrocytes in the CA3 and CA4 sub-regions of the hippocampus were counted. The area used for counting the cells was measured (SigmaScan Pro 5.5, SPSS) and was used for calculating cell density as cells/mm^2^ (8).

### Data analysis

The data were analysed using GraphPad Prism (v6.0). Two-way analysis of variance (ANOVA) was used for analysing the difference between the control and CML supplemented groups and different brain regions, with brain regions being treated as dependent factors. The specific changes in the brain regions were analysed using a multiple comparisons procedure (Sidak's) where an overall difference between the groups was identified. The difference of dopamine ratios between the CML and control supplemented groups was analysed using unpaired *t*-tests. The correlation between synaptophysin staining and our previously published MWM data (5) was also analysed using Pearson tests. The data are presented as mean±standard error of the mean (SEM). A *p* value of less than 0.05 was considered to be significant.

## Results

### Synaptophysin

**Fig. 1:**
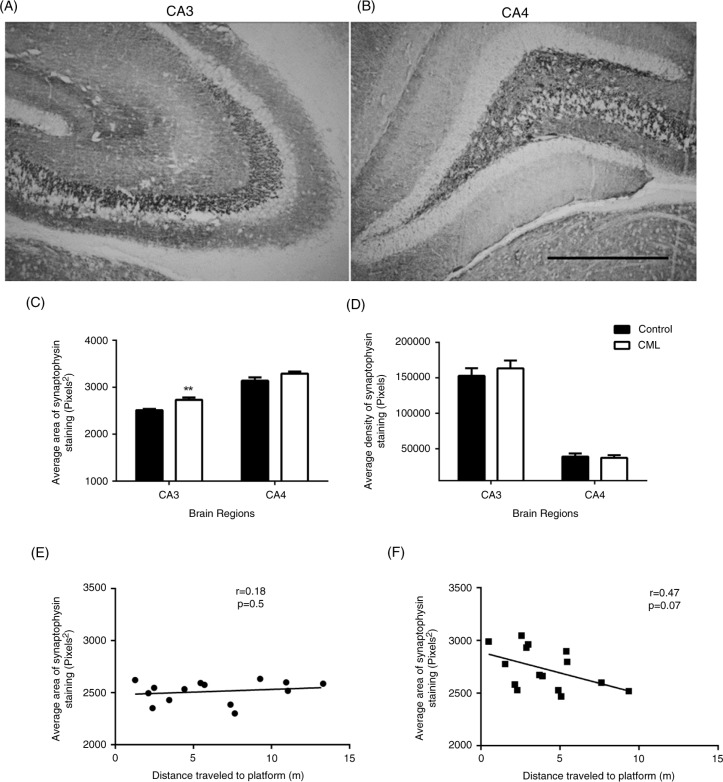
Changes in synaptophysin in the CA3 and CA4 sub-regions of the hippocampus. Representative images showing synaptophysin staining in the CA3 (A) and CA4 (B) hippocampal regions. The average area (C) and the average density (D) of synaptophysin staining in the CA3 and CA4 hippocampal regions of control (closed bars, *n*=15–16) and CML-supplemented (open bars, *n*=15–16) rats are shown. The increased area of synaptophysin staining in the CML-supplemented group (F, black squares) trended towards a correlation with the distance travelled to the platform of the MWM in day 2 of acquisition training (Supplementary Fig. 1). There was no correlation in the control group (E, black circles). Data are presented as mean±SEM, **p<0.01, scale bar=100 µm.

Synaptophysin staining was densely distributed within different sub-regions of the hippocampus, particularly in the CA3 (Fig. 1A) and CA4 (Fig. 1B) sub-regions. The microscopy images were representative of synaptophysin staining in the mossy fibres of the CA3 sub-region, and the CA4 sub-region, where it was defined in the area between the dorsal and ventral horns of the dentate gyrus. We evaluated both the average area (Fig. 1C) and the average density (Fig. 1D) of the staining in the sub-regions of the hippocampus.

Two-way ANOVA showed that the average area of staining was significantly different between the two regions (F[2,81]=84.55, *p*<0.001) and between the two groups (F[1,81]=7.95, *p*=0.0076), with a moderate interaction between the brain regions and the treatment groups (F[2,81]=4.46, *p*=0.014, Fig. 1C). *Post-hoc* tests showed that the average area of synaptophysin staining was significantly larger in the CA3 sub-region of CML group (*p*<0.01, *n*=16) compared to the control group (*n*=15, Fig. 1C). The increase in the average area of staining in the CA4 sub-region of the hippocampus of CML group was not significant (*p*=0.07, Fig. 1C). The average density of synaptophysin staining was similar between the two groups and two brain regions (Fig. 1D).

We also analysed the correlation between the area of synaptophysin in the CA3 sub-region of the hippocampus and the distance travelled to the platform in the MWM, which was significantly shorter in the CML-supplemented group than in the control group (*p*<0.05, *n*=16, Supplementary Fig. 1) (5). The shorter distance travelled to the hidden platform indicates the greater ability in learning. The average area of synaptophysin staining in the CA3 sub-region was trended towards a correlation with the distance travelled to the platform in the CML group (*p*=0.07, *r*=−0.47, Fig. 1F), but not in the control group (*r*=0.18, *p*=0.5, Fig. 1E).

### Tyrosine hydroxylase (TH)

**Fig. 2:**
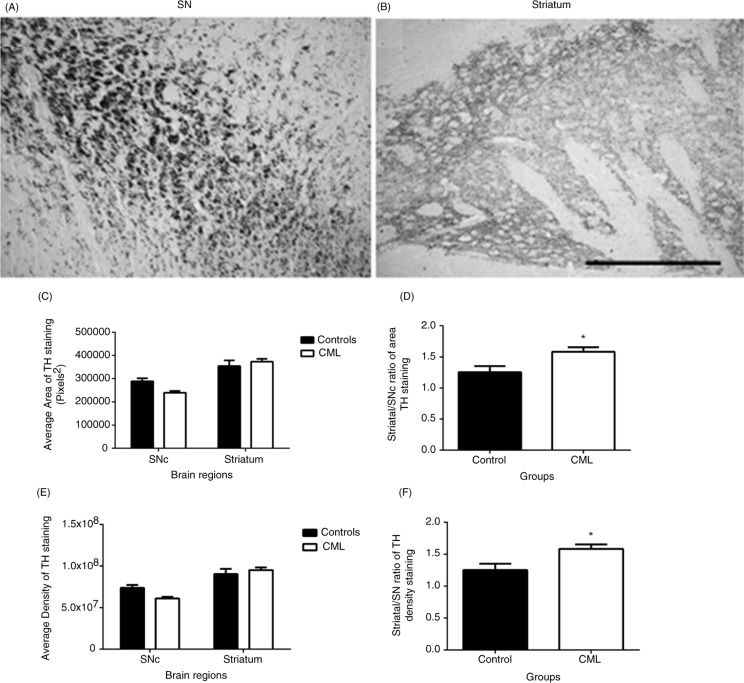
Changes in TH staining in the SNc and the striatum. Representative images showing TH (×20) staining in the A9 region of the SNc (A) and in the striatum (B) of rats. The average area of TH staining in the SNc and the striatum (C), the striatum/SNc ratio of the area of TH staining (D), the average density of TH staining in the SNc and the striatum (E), and the striatum/SNc ratio of the density of TH staining (F) of control (closed bars, *n*=15–16) and CML-supplemented (open bars, *n*=15–16) rats are shown. Data are presented as mean±SEM, *p<0.05, scale bar=100 µm.

Figure 2 shows microscopy images of TH staining in the SNc (Fig. 2A) and the striatum (Fig. 2B). The majority of TH staining in the SNc was associated to the process/axons of dopamine neurons and that in the striatum was located at the terminals of the dopamine neurons. To compare the association of dopamine immunoreactivity between the SNc and the striatum, TH staining was measured in the same sections, containing both SNc and striatum.

The average density and the average area of TH staining were evaluated in the A9 region of the SNc and in the striatum of both the control group (*n*=14) and the CML-supplemented group (*n*=15, Fig. 2C–F). Two-way ANOVA revealed a significant difference between the two brain regions in both the average area (*F* [1,54]=41.6, *p*<0.0001, Fig. 2C) and the average density (*F* [1,54]=41.01, *p*<0.0001, Fig. 2E) of TH staining, no differences between the two groups (*p*=0.3) with a moderate interaction between the brain regions and the groups (*F* [1,54]=4.8, *p*=0.03). To compare the relative changes, we also evaluated the striatal/nigral ratio of the TH staining. The striatal/nigral ratios of the average area and the average density were significantly increased in the CML group compared with the control group (*p*<0.05, Figs. 2D and F).

### Glial fibrillary acidic protein (GFAP)

**Fig. 3:**
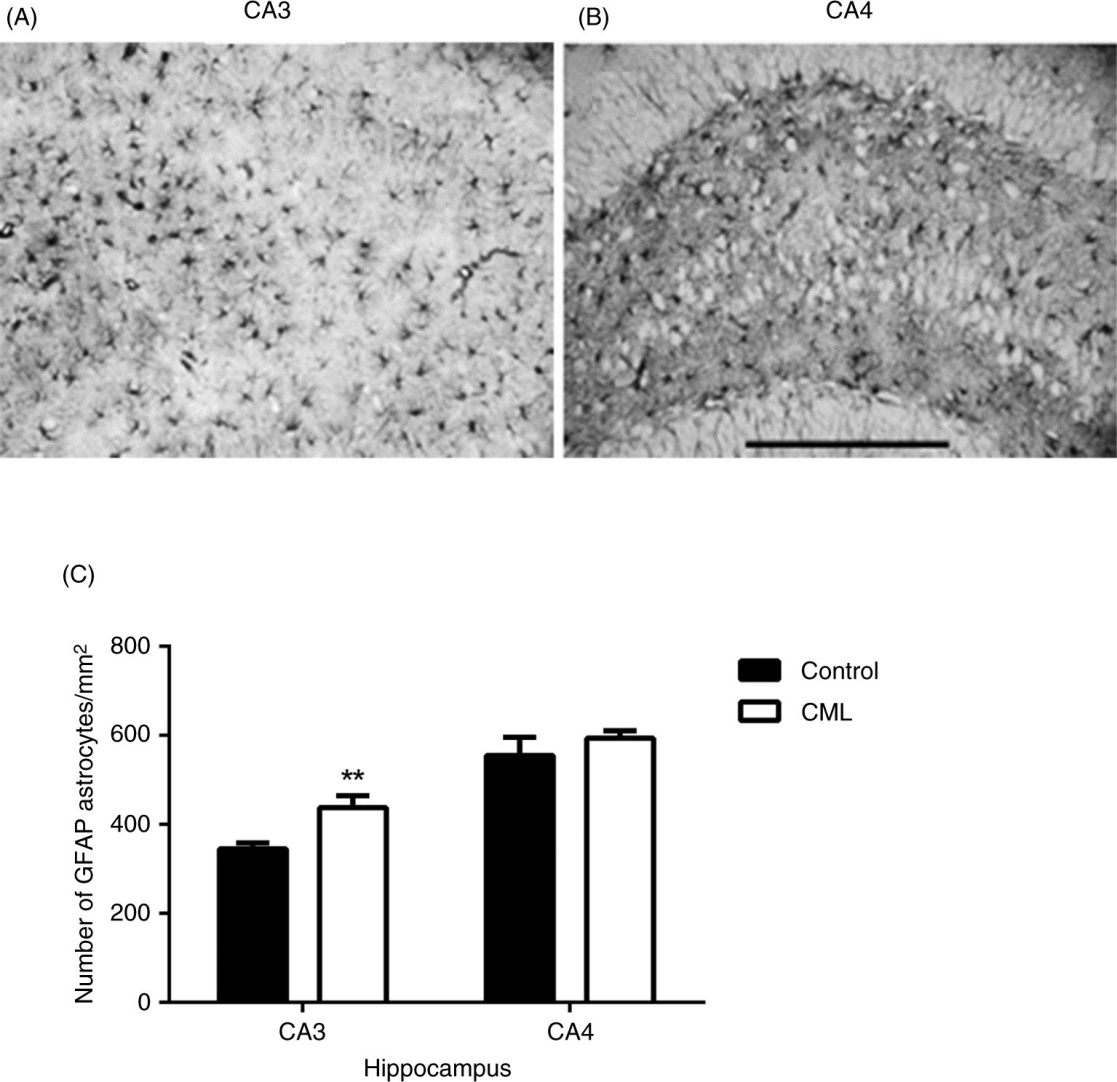
Changes in astrocytes in the hippocampus. Representative images showing GFAP-positive astrocytes in the CA3 (A) and CA4 (B) sub-regions of the hippocampus. The density of astrocytes in the CA3 sub-region of the hippocampus was significantly increased in the CML-supplemented group compared with the control group (C, *p*=0.0022, *n*=11–12). There was no change in astrocyte density in the CA4 sub-region of the hippocampus. Data are presented as mean±SEM, **p<0.01, scale bar=100 µm.

Figure 3 shows microscopy images of GFAP-positive astrocytes in the CA3 (Fig. 3A) and CA4 (Fig. 3B) sub-regions of the hippocampus. There was no hypertrophy morphology of the GFAP-positive astrocytes in any of the brain regions across both treatment groups. The density of GFAP astrocytes was used for the comparison. Two-way ANOVA showed a significant difference between the two groups (*F* [1,36]=10.6, *p*=0.002, *n*=7–12). There was no difference between the brain regions and no interaction between the groups and the brain regions. For the CML-supplemented group, *post-hoc* analysis showed a significant increase in GFAP astrocytes in the CA3 sub-region of the hippocampus (*p*<0.01), but not in the CA4 sub-region (Fig. 3C).

### Glutamate receptor-1 (GluR-1)

Neither the density nor the area of staining of GluR-1 was different between the control group and the CML-supplemented group in any of the brain regions measured (Table 1).

**Table 1:** Efficacy of CML supplementation on GluR-1, blood vessels, and MBP

Markers	Parameters measured	Regions	Controls (mean±SEM)	CML (mean±SEM)
GluR-1	Average area (pixels^2^)	Hippocampus	3,212±96 *n*=14	3,068±55 *n*=14
		Striatum	3,554±27 *n*=13	3,505±24 *n*=13
	Average density (pixels)	Hippocampus	291,956±17,566 *n*=14	307,382±16,474 *n*=14
		Striatum	248,696±9,185 *n*=13	229,874±6,158 *n*=13
Vessels	Total length (mm)	Hippocampus	2,505±203 *n*=14	2,386±136 *n*=15
		Striatum	1,850±202 *n*=13	1,692±130 *n*=15
	Total number (vessels/mm^2^)	Hippocampus	215±13 *n*=14	216±2 *n*=15
		Striatum	167±10 *n*=13	163±4 *n*=15
MBP	Average density	Hippocampus	163±1 *n*=16	161±4 *n*=15
		Striatum	170±2 *n*=16	171±4 *n*=15

### Vascular

Neither the total length nor the total number of vessels was different between the control group and the CML-supplemented group in any of the brain regions measured (Table 1).

### Myelin basic protein

Two-way ANOVA did not show any differences in MBP between the two treatment groups or in any of the brain regions examined (Table 1).

## Discussion

Overall, we found that long-term supplementation with CML during early brain development moderately promoted synaptic plasticity by showing a broader area of synaptophysin and elevated astrocytes in the hippocampus, as well as altered dopamine neuroplasticity in the striatum. CML supplementation did not alter myelination and vessel density.

Synaptophysin is an integral membrane protein of synaptic vesicles. As the expression of synaptophysin indicates non-specific changes in synaptic activity at pre-synaptic locations (20), it has been widely used as a marker of synaptic plasticity (21–25), including dietary-associated plasticity in the hippocampus (26). The improved synaptic plasticity after CML supplementation appeared to be more specific to the changes of the area of staining but not the density within the area. Changes in synaptophysin can often be evaluated in terms of the density and the area of reactivity (13). While changes in density reflect the vesicle activity within the area, in which synaptic activity may be reduced (12, 27). Large area of synaptopysin staining may suggest broader, perhaps new, synaptic connections. The immunoreactivity of synaptophysin has commonly been used for indicating hippocampal plasticity associated with MWM performance (13, 26). We have previously reported that CML supplementation in rats results in improved learning in the MWM and better performance in novelty recognition, compared with control rats. Although there was no correlation in the control group (Fig. 1E), the improved performance in the MWM (Supplementary Fig. 1) may be associated with broader synaptic activity (Fig. 1F).

We have recently shown that memory improvement after long-term supplementation with a different form of CML (CML concentrate) was associated with enhanced synaptophysin density in the CA3 sub-regions of aged rats (12), in which synaptic function was reduced in the control group of aged rats (12). The increase in density could have been a result of preventing/restoring the loss of synaptic function, rather than promoting synaptogenesis. As synaptogenesis is a major part of brain development in the postnatal period, the broader synaptic activity may be the changes more specific to brain development. The changes in synaptophysin appeared to be specific to the CA3 sub-region of the hippocampus, a region that plays a critical role in the encoding of new spatial information (7). Compared to the aged rats, which have clear deficits in memory, the efficacy of nutritional intervention on young normal rats appears to be more moderate in both functional outcome and biological improvements (12). The efficacy of CML supplementation in the normal rat therefore raises the important question of treatment effects in the setting of learning deficits, such as those evidenced by foetal growth retardation, and warrants further investigation.

Complex lipids have previously been implicated in improvements in neuroplasticity (12, 28). Phosphatidylserine has been perceived to participate in the activation of genes that are required for long-term neuroplasticity (28). Gangliosides, which are bioactive ingredients in the CML supplement, have previously been suggested to enhance neuroplasticity in the CA3 hippocampal region. For example, treatment of hippocampal slices with gangliosides GM1 and GD1b improves the synaptic plasticity of pyramidal neurons in the CA3 region (29).

Apart from neurons, a large part of the TH staining in the SNc is located to neuronal dendrites and axons, which project into the striatum, in which terminal TH staining is often used for evaluating dopamine outputs (30). To compare differences between neuronal and terminal staining, we used sections that contained both regions. As the SNc used for evaluation was more anterior to the centre of the nuclei, the staining was mainly of neuronal dendrites and axons, with very few neurons. We did not see any significant difference in TH reactivity between the two groups, particularly in the striatum, despite a possible lower TH staining in the SNc of the CML-supplemented group. As terminal TH staining would be more representative of dopamine function, these data suggest that CML supplementation did not alter dopamine function.

To evaluate the potential effect on neuroplasticity in the nigral–striatal dopamine pathway, we analysed the terminal/neuronal staining ratio. The ratio values were greater than 1 in all rats, indicating that the density of TH staining was greater in the striatum than in the SNc. CML supplementation significantly increased the relative value of dopamine output over neuronal staining in both the density and the area of staining (Fig. 2). Even though these changes were not related to dopamine output, the data support the efficacy of CML in altering synaptic plasticity. Without counting the number of dopamine neurons in the SNc, the relatively lower staining in the SNc may not necessarily indicate fewer dopamine neurons, as TH is generally found to be more abundant in the neuronal terminals in the striatum than in the neuronal soma and processes in the SNc (31). We have recently reported that improvements in the memory following supplementation with CML concentrates in aged rats was mediated through prevention and/or restoration of nigral–striatal dopamine depletion (12), a pathway that plays a key role in learning and memory (11). However, there is no association between the changes in TH and the performance in the MWM (data not shown).

Astrocytes are essential for, and play a critical role in, synaptic genesis and plasticity (14, 32, 33), for example, the formation and stabilisation of synaptic connections during brain development (34). A reduction in astrocytes is associated with the loss of pre-synaptic plasticity indicated by synaptophysin (12), but not of post-synaptic density protein PSD95, a marker for post-synapses in rats with age-related cognitive decline (35). Morphologically, the astrocytes did not show any sign of hypertrophy, suggesting that the reactivation of astrocytes was not an immune response. CML supplementation elevated the density of astrocyte staining in the CA3 sub-region of the hippocampus, but not in the CA4 sub-region (Fig. 3), a brain-region-dependent effect that is similar to synaptogenesis. Thus, the elevated levels of astrocytes in the CA3 sub-region of the hippocampus may have a role in forming new synapses, therefore providing indirect evidence for improved synaptic plasticity after CML supplementation.

Even though phospholipids and gangliosides are critical components of the myelin sheath (17), supplementation with CML did not alter the degree of myelination in the brain regions involved in processing learning and memory. Similarly, supplementation during brain development did not change vascular density.

## Conclusion

Overall, these data suggest that CML supplementation during development moderately promoted synaptic plasticity in the CA3 sub-region of the hippocampus and altered nigral–striatal dopamine pathway in young normal rats. CML supplementation did not alter white matter development or vascular remodelling.

## Supplementary Material

Supplementation with complex milk lipids during brain development promotes neuroplasticity without altering myelination or vascular density
