# Supplementary material for: Supplementation with complex milk lipids during brain development promotes neuroplasticity without altering myelination or vascular density
Source: Food Nutr Res. 2015 Mar 27;59:10.3402/fnr.v59.25765. doi: 10.3402/fnr.v59.25765 (PMC4377325; doi:10.3402/fnr.v59.25765)
Supplement: Supplementation with complex milk lipids during brain development promotes neuroplasticity without altering myelination or vascular density [file FNR-59-25765-s001.pdf]

Supplementary Figure 1

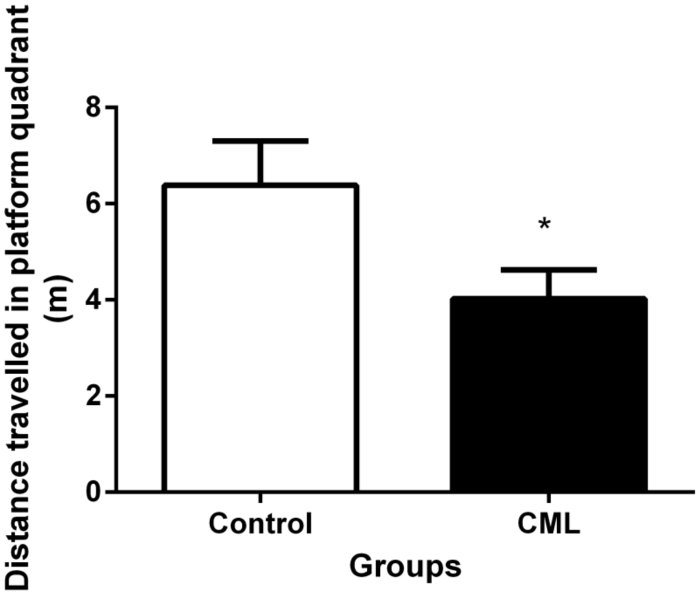

Supplementary Figure 1 shows that distance travelled for locating the platform was significantly reduced in the rats with CML supplementation.
